# Treatment of Twin Anemia Polycythemia Sequence with Donor Transfusion and Partial Recipient Exchange Transfusion: Procedural Considerations and Outcomes

**DOI:** 10.3390/jcm13175068

**Published:** 2024-08-27

**Authors:** Camille F. Shantz, Mara Rosner, Michelle L. Kush, Jena L. Miller, Ahmet A. Baschat

**Affiliations:** Johns Hopkins Center for Fetal Therapy, Department of Gynecology and Obstetrics, Johns Hopkins School of Medicine, Baltimore, MD 21287, USA

**Keywords:** twin anemia polycythemia sequence, fetal partial exchange transfusion, fetal transfusion, fetal therapy, monochorionic twins

## Abstract

**Background:** Intrauterine transfusion (IUT) of the donor and partial exchange (pET) of the recipient is a temporizing treatment for pregnancies with Twin Anemia Polycythemia Sequence (TAPS). We aimed to provide a detailed description of the procedural approach and outcomes for sequential donor IUT and recipient pET in TAPS. **Methods:** Retrospective study of spontaneous TAPS referred to the Johns Hopkins Center for Fetal Therapy treated with donor IUT followed by recipient pET utilizing a double-syringe setup. Procedural characteristics and outcomes as well as the accuracy of existing transfusion formulas were analyzed and compared with the literature. **Results:** 5 of 78 patients with spontaneous TAPS underwent a total of 19 combined IUT/pET procedures (median first procedure to delivery interval 5.6 weeks [interquartile range IQR 1.9–6.0]). One pET was stopped due to fetal deceleration. The patients were delivered at 33.0 weeks [IQR 31.9–33.3] with two survivors and no neonatal transfusion requirements. The IUT volume was 48 mL [IQR 39–63 mL] and the pET volume was 32 mL [IQR 20–50], utilizing aliquots of 5–20 mL for the latter (*p* = 0.021). For the IUTs, the assumption of a fetal blood volume below 150 mL/kg underestimated the required transfusion volume. For the pETs, all formulas required adjustment of the dilution volume based on bedside testing (*p* < 0.05 for all). **Conclusions:** Donor transfusion followed by partial exchange in the recipient can prolong pregnancy in spontaneous TAPS and obviate the need for neonatal transfusion. A double-syringe setup facilitates efficient saline exchange. Because the accuracy of volume formulas is limited, bedside testing is recommended to achieve the target hemoglobin.

## 1. Introduction

In Twin Anemia Polycythemia Sequence (TAPS), unequal red blood cell passage across intertwin anastomoses of the monochorionic twin placenta predisposes the donor to anemia and the recipient to polycythemia. Unlike Twin-to-Twin Transfusion Syndrome, the placental vascular anastomoses responsible for TAPS may not be fetoscopically accessible, necessitating a wider range of management approaches [1]. Temporizing treatment of both twins in TAPS requires intrauterine transfusion (IUT) of the anemic donor and hemodilution of the polycythemic recipient, achieved through a partial exchange transfusion (pET). This combined treatment is rarely described, potentially due to lower utilization or procedural complexity [2].

In contrast to ultrasound guided IUTs, which are among the widest offered fetal interventions with a standardized procedural approach, fetal pET is infrequently performed (Table 1) [3,4]. Historically, fetal exchange transfusions were performed prior to the 1990s for anemic fetuses with immune hydrops. These procedures intended to exchange the blood volume for donor cells compatible with the maternal blood type to decrease the risk of ongoing hemolysis [5,6,7]. While these reports describe the basic technique of sequential removal and transfusion of blood aliquots, these procedures were not intended to achieve hemodilution to a specific target hemoglobin. Robyr et al. was the first to describe partial fetal hemodilution in the polycythemic recipient twin for postlaser TAPS [8]. Subsequent reports that described sequential IUT and pET varied in detail and the procedural approach (Table 1) [9,10,11,12,13]. It was the goal of this study to present our outcomes with a detailed description of the procedural approach and success with sequential donor transfusion and recipient exchange in TAPS and compare our findings with the existing literature.

## 2. Materials and Methods

We retrospectively reviewed all monochorionic twins referred to the Johns Hopkins Center for Fetal Therapy between July 2014 and September 2023. Patients underwent a detailed ultrasound assessment including an anatomic survey, fetal echocardiography, fetal biometry, amniotic fluid volume, and fetal and maternal Doppler studies, using high-resolution ultrasound (GE Voluson E10). The diagnosis of TAPS was made when the middle cerebral artery peak systolic velocity (MCA PSV) in the donor exceeded 1.5 multiples of the median (MoM) and was below 0.8 MoM in the recipient. Patients meeting the criteria for TAPS were staged by severity [14]. Patients were offered Solomon laser, IUT and pET, selective twin reduction, expectant management or pregnancy termination based on the severity of the condition and the feasibility of surgical treatment. This study was focused on patients receiving IUT with pET as the primary treatment.

The IUT and pET procedure was performed in the ultrasound office or in labor and delivery after viability. We always transfused the donor twin first followed by the partial exchange of the recipient. This sequence was chosen to treat the anemic fetus first and for the potential advantage of addressing any intraprocedural red cell transfer to the recipient by adjusting the hemodilution volume. Continuous guidance with a 2–6 MHz high frequency ultrasound transducer was utilized throughout the procedure (E10, GE Healthcare, Wauwatosa, WI, USA). Fetal paralysis was administered first by ultrasound guided intramuscular or intravascular fetal injection of rocuronium (0.5–1 mg/kg sonographically estimated fetal weight). All IUT and pET procedures are performed with a 20-gauge needle utilizing placental cord insertion, the free loop or intrahepatic umbilical vein for access based on operator preference. Bedside point of care testing of fetal blood samples was performed to verify that target hemoglobin values were achieved (HemoCue HB 201 DM analyzer; HemoCue USA, Brea, CA, USA).

**Figure 1:**
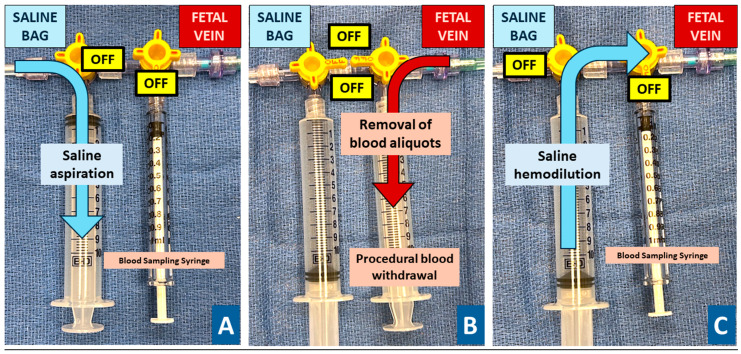
Image of partial exchange transfusion setup. Legend: The figures illustrate the procedural setup for fetal exchange transfusion using a sterile connection and tubing to connect to the saline bag. Two three-way stopcocks are positioned serially, and a short tubing connects to the 20-gauge needle once the fetal vein has been accessed. For priming of the syringe with saline aliquots, the access to the fetal vein and blood sampling port is turned off (**A**). This allows repeated priming of the syringe while closing off access to the fetal circulation. Once an opening blood sample has been obtained, switching the stopcocks to the “off” position toward the saline reservoir (**B**) allows for the removal of fetal blood aliquots and intraprocedural blood sampling. For the administration of saline aliquots, the access to the saline bag and blood sampling syringed are turned to the “off” position (**C**).

The anemic donor was transfused based on the opening hemoglobin with O-Rhesus negative blood with a hemoglobin concentration of 25 mg/dL at a rate of 1–5 cc/min with the goal of achieving a normal hemoglobin value. After successful vascular access, an initial blood sample was taken to determine the opening fetal hemoglobin value by bedside testing. Based on the opening hemoglobin value, the transfusion volume was estimated based on the estimated fetal weight [15]. During the fetal blood transfusion, the position of the needle tip, fetal heart rate and intravascular flow of transfused blood was monitored by ultrasound. After the anticipated blood volume was administered, a preliminary closing sample was obtained for bedside testing. If the hemoglobin value was lower than expected, additional blood was administered until the desired fetal hemoglobin was verified on follow up bedside testing. Once the donor transfusion was completed, blood sampling was performed of the recipient and paralysis was administered if fetal movement was still present. After successful vascular access, an opening sample was obtained for bedside testing of the hemoglobin. Then, a double-syringe setup was attached to the needle that allowed alternating blood withdrawal and saline infusion without detachment (Figure 1). Guided by the opening hemoglobin value, blood was withdrawn in 5–20 cc aliquots followed by the infusion of matching aliquots of 0.9% sterile saline solution with the goal of diluting it to a high-normal hemoglobin concentration. The exchange volume was estimated using formulas originally published for partial volume exchange in neonates and further adjusted, guided by intraprocedural samples and bedside testing [16]. After the anticipated blood volume was withdrawn and saline was administered, a preliminary closing sample was obtained for bedside testing. If the hemoglobin value was higher than expected, additional blood was withdrawn and exchanged with normal saline to reach the desired fetal hemoglobin value verified by bedside testing. For all procedures, the opening and closing blood samples were also analyzed in the central laboratory with additional determination of the fetal hemoglobin content by Kleihauer–Betke (KB) testing. Patients were discharged for outpatient ultrasound monitoring after the procedure, after the return of fetal activity and a reassuring fetal heart rate for procedures performed after viability.

We recorded pre-procedure characteristics including placental location, MCA Doppler for both twins, estimated fetal weights and TAPS stage. Procedural characteristics included gestational age, maternal anesthesia, fetal paralysis and tocolytic treatment. The details on needle gauge, fetal vascular access, procedure duration, and the opening and closing hematocrit, hemoglobin, and KB values for each fetus as well as any procedural complications were recorded. For transfusions, we noted the administered blood volume, and for pETs, the aliquot size of the removed blood and administered saline. Pregnancy outcomes were ascertained after delivery from the respective delivery sites.

For blood transfusions, we compared the recorded transfusion volume and observed changes in donor hemoglobin to the estimated transfusion volume based on published formulas [9,15,17,18,19]. The difference of the observed to expected transfusion volume was determined to quantify the deviation for each of the above formulas. The same approach was taken for the volume of blood partially exchanged with normal saline during recipient exchange transfusions [9,16,20]. The difference in the observed to expected exchange volume was determined to quantify the deviation for each of the above formulas. We used descriptive statistics for pre-, intraprocedural and outcome details. Comparisons between observed to expected transfusion and exchange volumes used Mann–Whitney U or Kruskal Wallis tests as appropriate. Pooled statistics of this case series and the published literature are presented for datapoints where the corresponding details could be ascertained (Table 1). Statistical analyses were performed via IBM SPSS Statistics version 26.02 (IBM Corp., Armonk, NY, USA). A *p*-value less than 0.05 was considered significant for all comparisons.

## 3. Results

Five of 78 patients (6.4%) presenting with spontaneous TAPS were treated with concurrent donor transfusion and pETs of the recipient. The median time at diagnosis was 27.6 [interquartile range (IQR) 25.1–28.9] weeks and the majority had stage II TAPS. The cohort underwent a total of 20 IUTs; in one procedure, only fetal blood sampling was performed on the TAPS recipient (Table 1). Two of five patients had anterior placentas, and the median gestational age at first procedure was 28.3 [IQR 27.1–30.0] weeks. All procedures were performed under local anesthesia, with 3 of 20 (15%) with supplementary maternal intravenous sedation. Most patients received a tocolytic pre-procedure (80%). The median interval between serial IUTs with pET was 8 [IQR 7–10] days and the interval from the last procedure to delivery was 5.7 [IQR 1.3–6.9] weeks. The median gestational age at delivery was 33.0 [IQR 31.9–33.3] weeks, a median of 5.6 [IQR 1.9–6.0] weeks after the first procedure.

Three IUT procedures required a second attempt to achieve vascular access. One patient underwent IUT with pET and an amnioreduction in the setting of stage I TTTS at 29.1 GA. In one IUT, an additional intraperitoneal transfusion was performed. During one pET, procedure fetal heart rate decelerations occurred after withdrawal of 5 cc of blood in a recipient with 54.3% hematocrit. After cessation of the procedure and successful intrauterine resuscitation no subsequent procedures were attempted, and the patient subsequently delivered two weeks later at 34.3 GA with two live births. We encountered no other complications.

Although the median number of samples during procedure types was comparable, three or more samples were utilized in more than 30% of pETs (Table 2). A median of 48 [IQR 39–63] cc of blood was transfused to the anemic fetus in IUTs, and 32 [IQR 20–50] cc of blood was exchanged for 32 [IQR 15–20] cc of saline in pETs. The aliquot size ranged from 5 cc to 20 cc and varied during the procedure with the most common aliquot size being 10 cc (Table 2). The magnitude of hemoglobin change was greater for transfusions (7.2 [IQR 5.25–8.27] g/dL versus 2.5 [IQR 1.4–3]) g/dL than partial exchanges (Mann–Whitney U, *p* < 0.05).

Following the transfusion treatments, the delivery indications were unrelated to TAPS and all patients had concordant MCAs at their last ultrasound prior to delivery. Three patients underwent planned, elective cesarean sections; one patient underwent delivery for pre-eclampsia; and one patient underwent delivery due to progressing Twin to Twin Transfusion syndrome with anhydramnios and abnormal Dopplers. Every patient had two live births with neither baby requiring neonatal intervention for anemia or polycythemia.

In general, the IUT volumes were higher than the pET volumes (*p* = 0.021, Table 2). Formulas assuming a blood volume of 150 mL per kg sonographically estimated fetal weight underestimated the transfusion non-significantly (*p* = 0.258, Table 3). A lower estimated fetal blood volume underestimated the transfusion volume to achieve the target hemoglobin by 15 [IQR 13.5–21.5] ml (*p* = 0.002, Table 3). For pETs, the removed volume aliquots required to achieve the desired hemoglobin value differed from the predicted values for all formulas. Formulas based on a blood volume of 150 mL/kg fetal weight overestimated the volume of blood to be removed by 8.5 milliliters [IQR 11.6 to 6.84], while lower blood volume assumptions underestimated the amount by 6.2–22.3 milliliters (*p* < 0.05 for all, Table 3).

A literature review identified five manuscripts describing variable procedural details on nine patients with postlaser or spontaneous TAPS undergoing a total of 16 IUTs with pET [8,9,10,11,12]. Combined with the current series, diagnosis was at a median gestational age of 27.1 [IQR 25–27.6] weeks and the first treatment was instituted at 27.3 [IQR 25.4–28.6] weeks. With a median interval of 4.7 [IQR 2.1–5.6] weeks after the initiation of transfusions, patients delivered at 31.8 [IQR 30.0–33.3] weeks gestation (Table 1).

## 4. Discussion and Conclusions

Intrauterine transfusion of the anemic donor twin and partial exchange transfusion of the polycythemic recipient is an infrequently reported temporizing management for patients with twin anemia polycythemia sequence. With most patients presenting in the late second trimester, this management approach potentially delayed delivery by almost 5 weeks across all reported cases, with no additional postnatal transfusion required in the five cases presented in this series. Our procedural approach of sequentially targeting the anemic donor and then the polycythemic recipient twin was successful in almost all procedures. While formula estimation of the anticipated transfusion volume worked well for the donor, the dilution volumes for the recipient were inadequately predicted by formulas derived from neonatal pETs. This stresses the importance of bedside testing to achieve the target hemoglobin values during pETs.

Because TAPS has differing presentations, management approaches can vary [1]. Prior reports on exchange transfusion treatments have not described the procedural approach with the same degree of detail as presented here. We used larger aliquots and perform IUT prior to pET, addressing the more compromised donor first [21]. While performing IUT only will address the more clinically pressing needs of the anemic, donor fetus, an additional recipient exchange allows for monitoring of the hemoglobin values of the polycythemic fetus and dilution of the blood volume that may have passed over during the donor transfusion. KB tests in the opening sample of the recipient suggests that this indeed does happen. While our sample size is too small to analyze for the dynamics of the intraprocedural blood transfer, additional measurements of the recipient KB following donor transfusion may provide insight into the impact of donor IUT on recipient polycythemia. This testing could also be utilized clinically to identify cases with accelerated intertwin blood transfer that are unlikely to benefit from fetal transfusions as the primary management strategy.

While the ability to perform bedside hemoglobin testing of samples may be advantageous to capture intertwin transfusion of donor blood to the recipient, it is important during both interventions in the combined procedure. All published IUT and pET formulas are modifications of the neonatal formula, differing with their evaluation of circulating blood volume per kilogram of estimated fetal weight [20]. The placental contribution to fetal circulating blood volume has been studied [22,23], but the intraprocedural transfusion of blood between fetuses and the monochorionic physiology may further complicate formulaic estimation of the transfusion and exchange volumes. Ultimately, our practice is to use formulas to guide the beginning of a procedure and then rely on bedside testing of intraoperative sampling to adjust the transfusion and exchange volumes.

Our study is limited by a small number of cases. Our cohort is also difficult to compare to the literature because we report spontaneous TAPS cases, which may have better outcomes than postlaser TAPS [4]. Furthermore, while the bedside hemoglobin and hematocrits presented in our analysis inform procedural decision-making, they may be less accurate than laboratory testing. Finally, our comparison to the existing literature is limited by the lack of published detail on previously reported cases. While pET was initially described in 2006 by Robyr, the absence of any description precludes us from including these cases in Table 1 [8].

Our study benefits from the largest number of procedures detailed so far, doubling the case size in the existing literature, and our thorough description of our procedural approach. The intraprocedural sampling hematocrits and KB values contribute to investigations of TAPS disease processes; our outcomes encourage clinicians to consider pET when treating TAPS with IUT; and our procedural description aids other groups in refining their pET procedural approach.

We recognize that currently there is insufficient information to provide a clear indication when pETs should be performed in TAPS. A neonatal partial exchange is a more extensive procedure and the fact that none of our patients required these could be considered a benefit. Overall, the hemoglobin correction achieved by pETs is less than that achieved by transfusions and further studies will need to demonstrate if the procedural risks are offset by the fetal and neonatal benefits.

In conclusion, we demonstrate that the double procedural setup can be effectively performed to prolong pregnancy. While we do not offer an analysis of serial IUTs without pET, the concordant MCAs prior to delivery, our neonatal outcomes, and previous modeling by Slaghekke et al. suggest that our combined approach was effective in preventing serial IUTs from exacerbating the polycythemia of the recipient twin [13]. Combined pET/IUT for TAPS requires further study but is a promising temporizing treatment for prolonging pregnancy and mitigating poor neonatal outcomes.

## Figures and Tables

**Table 1:** Reported Cases of Concurrent IUT/pETs for TAPS.

Reference	Stage	Dx GA	GA at 1st Procedure	IUT/pET Procedures	1st Rx to Delivery Interval (Weeks)	Delivery GA	TAPS Rec BW (g)	TAPS Don BW (g)	TAPS-Related Neonatal Complications
INDIVIDUAL CASES REPORTED IN THE LITERATURE									
Bahtiyar [9]	IV II II	26.1 27.6 24.6	26.3 27.7 24.9	1 2 1 *	1.0 2.7 11.3	27.3 30.4 36.2	NR NR NR	NR NR NR	2 Partial exchanges 1 Partial exchange -
Genova [10]	IV+ II+ II+	23.6 27.1 27.1	23.7 27.3 28.3	4 1 * 2	4.1 5.4 2.9	27.8 32.7 31.2	1235 1615 1520	980 1664 1759	Donor cerebral injury, NND Partial exchange -
Gucciardo [11]	I	28.9	29.0	1++	5.3	34.3	1805	1525	-
Moaddab [12]	IV+	25.0	25.1	1 *	0.9	26	1049	1145	Partial exchange, Double NND
Slaghekke [13]	NR+	NR	26.4	3	5.3	31.7	NR	NR	NR
CASES REPORTED IN THIS SERIES									
Case 1 Case 2 Case 3 Case 4 Case 5	IV II II II II	22.9 27.6 29.4 28.9 25.1	23.7 28.7 30.0 29.1 27.3	7 * 3 3 1 ** 4	9.4 5.6 1.9 0.7 6.0	33.1 34.3 31.9 29.9 33.3	1705 1880 1600 1335 1940	1690 1475 1235 910 2050	- - - - -

Legend: + = postlaser twin anemia polycythemia sequence; additional treatments performed: * additional donor transfusion only, ** additional amnioreduction, ++ additional fetoscopic laser performed. BW, birthweight; Don, donor; Dx, diagnosis; GA, gestational age; IUT, intrauterine transfusion; NND, neonatal demise; NR, not reported; pET, partial exchange transfusion; Rec, recipient; Rx, treatment; TAPS, twin anemia polycythemia sequence.

**Table 2:** Procedural details of donor transfusion and recipient partial exchange transfusions.

	Donor Transfusions (n = 20)	Recipient Partial Exchange (n = 19)
Estimated fetal weight target fetus (g)	1115 [875–1405]	1345 [1067–1555]
Pre-procedure MCA PSV MoM	1.67 [1.40–1.88]	0.82 [0.67–1.09]
Opening hemoglobin value (g/dL)	7.9 [5.4–9.8]	19.8 [18.0–20.8]
Opening Kleihauer–Betke (%)	50 [26.7–100]	73.5 [53.1–89.9]
Hydrops of target fetus	2 (10)	0 (0)
Paralysis of target fetus	20 (100)	17 (89.5)
Intravascular access Intrahepatic Free loop Placenta cord insertion	2 (10) 3 (15) 15 (75)	7 (36.8) 3 (15.8) 9 (47.4)
Procedure time (min) *	16 [13–21]	22 [15–30]
Number of access attempts 1 2	17 (85) 3 (15)	19 (100) 0 (0)
Number of samples after opening * 1 2 3 4 5 Median (range)	4 (20) 12 (60) 4 (20) 0 (0) 0 (0) 2 (1–3)	5 (26.3) 7 (36.8) 4 (21.1) 2 (10.5) 1 (5.3) 2 (1–5)
First aliquot size 5 10 15 20	- - -	3 (15.7) 13 (68.4) 1 (5.3) 2 (10.5)
Total blood/saline added *	48 [39–63]	32 [20–50]
Total blood removed *	-	32 [15–50]
Closing hemoglobin value (g/dL)	15.3 [14.5–16.0]	16.8 [16.4–18.4]
Closing Kleihauer–Betke (%)	16.8 [14.0–23.1]	78.2 [47.9–85.0]
Change in hemoglobin (g/dL) *	+7.2 [5.25–8.27]	−2.5 [−3.0–−1.4]
Post procedure MCA PSV MoM *	0.99 [0.83–1.12]	0.89 [0.82–1.03]
Last MCA PSV MoM before delivery	1.15 [1.11–1.18]	1.02 [0.94–1.12]

Legend: Data are expressed as number (percent) or median [interquartile range]. * one pET excluded due to early termination of the procedure; MCA, middle cerebral artery; MoM, multiples of median; pET, partial exchange transfusion; PSV, peak systolic velocity.

**Table 3:** Difference between formula-projected and actual transfusion volumes.

Formula	Reference	Median Difference From Actual Volume	*p*-Value
$VT=\left(EFW*82.5\right)\frac{\left(Hct_{f}-Hct_{i}\right)}{Hct_{d}}$	[18]	15 [13.5–21.5]	0.002
$VT=\left(EFW*150\right)\frac{\left(Hct_{f}-Hct_{i}\right)}{Hct_{d}}$	[8,14,16]	4.8 [−6.64–10.8]	0.258
$VT=\left(EFW*162\right)\frac{\left(Hct_{f}-Hct_{i}\right)}{Hct_{d}}$	[17]	2.0 [−11.0–8.4]	0.470
$VD=\left(EFW*90\right)\frac{\left(Hct_{i}-Hct_{f}\right)}{Hct_{i}}$	[15]	22.3 [12.6–36.03]	<0.001
$VD=\left(EFW*106\right)\frac{\left(Hct_{i}-Hct_{f}\right)}{Hct_{i}}$	[19]	6.2 [5.02–8.12]	0.001
$VD=\left(EFW*150\right)\frac{\left(Hct_{i}-Hct_{f}\right)}{Hct_{i}}$	[8]	−8.5 [−11.6–−6.84]	0.038

Legend: Data are expressed as median [interquartile range]. Projected volumes were calculated based on a transfusion unit hematocrit of 75%. Statistical comparisons by Mann–Whitney U testing. EFW, estimated fetal weight in kilograms; Hct_d_, hematocrit of transfused donor blood; Hct_f_, final hematocrit; Hct_i_, initial hematocrit; VD, volume of dilution; VT, volume of transfusion.

## Data Availability

The data are available upon reasonable request of the authors.
